# Shelf-Life Prediction of Shrimp Gravlax Using Machine Learning: Integrating Traditional Processing with AI Modeling

**DOI:** 10.3390/foods15101805

**Published:** 2026-05-20

**Authors:** Ozlem Emir Coban, Ilhan Firat Kilincer, Aniseh Jamshidi, Mehmet Zulfu Coban

**Affiliations:** 1 Department of Fish Processing Technology, Faculty of Fisheries, Firat University, Elazig 23119, Turkey; 2 Department of Digital Forensic Engineering, Firat University, Elazig 23119, Turkey; 3 Department of Fisheries, Faculty of Marine Natural Resources, Khorramshahr University of Marine Science and Technology, Khorramshahr 64199-34619, Iran; a.jamshidi1382@gmail.com; 4 Department of Food Processing, Keban Vocational Schools, Firat University, Elazig 23119, Turkey; mzcoban@firat.edu.tr

**Keywords:** gravlax, shrimp, vacuum-packaging, storage quality, machine learning algorithms

## Abstract

This study aimed to develop shrimp gravlax (*Penaeus japonicus*) as a ready-to-eat seafood product and to determine its shelf life. The product was prepared using a curing method and stored at 4 °C for 30 days. Quality changes were monitored at five-day intervals through analyses of TVB-N, TBARs, peroxide value, pH, water activity, total mesophilic aerobic bacteria, and total psychrophilic bacteria. Gradual shifts in quality parameters were observed during storage, with notable increases in TVB-N, lipid oxidation markers, and microbial counts. Sensory scores declined over time, yet the product remained acceptable until approximately day 25. These findings suggest that shrimp gravlax has a shelf life of around 25 days under the studied conditions. To support freshness evaluation, machine learning models including Support Vector Machine (SVM), K-Nearest Neighbors (K-NN), and Decision Tree (DT) were applied. After data augmentation and parameter optimization, the models achieved high classification performance, reaching up to 100% under optimized conditions. The classification outcomes aligned well with experimental observations, highlighting the potential of machine learning to strengthen shelf-life assessment when multiple quality indicators are considered together. Nevertheless, the models were developed under a single storage condition and focused on classification rather than time-series prediction. Further research using independent datasets and varied storage environments will be necessary to enhance model generalizability. In conclusion, shrimp gravlax can be regarded as a promising ready-to-eat product. Combining traditional processing methods with machine learning provides a practical and innovative approach to shelf-life evaluation in seafood systems.

## 1. Introduction

Seafood products are highly perishable due to their biochemical composition and susceptibility to microbial growth. Among them, shrimp is widely consumed because of its nutritional value and desirable sensory properties [1]. However, its relatively short shelf life remains a major challenge, particularly during storage and distribution.

Gravlax is a traditional curing method commonly applied to fish, especially salmon, using a mixture of salt, sugar, and various seasonings. In addition to improving sensory characteristics, this process can help extend shelf life by reducing water activity and slowing down microbial growth [2,3]. While gravlax has been widely studied in fish products, its application to shrimp has received less attention and still requires further investigation [3].

The evaluation of shelf life in seafood products generally involves monitoring several quality parameters that reflect chemical and microbiological changes during storage. Total volatile basic nitrogen (TVB-N) is commonly used to assess protein degradation and spoilage development. Lipid oxidation, which is another major factor affecting product quality, is typically evaluated using peroxide value (PV) and thiobarbituric acid reactive substances (TBARs), representing primary and secondary oxidation products, respectively. In addition, pH changes are often associated with the formation of alkaline compounds during storage, while water activity (aw) plays an important role in controlling microbial growth and overall product stability. Since these parameters are influenced by both processing and storage conditions, evaluating them together provides a more comprehensive understanding of shelf life [4,5,6,7].

In recent years, machine learning (ML) approaches have been increasingly used in food science for quality evaluation and shelf-life prediction. These methods allow multiple variables to be analyzed simultaneously and can reveal relationships that may not be easily detected using conventional approaches. In seafood systems, parameters such as TVB-N, oxidation indicators, and microbial counts have been reported to be particularly effective inputs for predictive models [4,5,8,9].

Although the machine learning algorithms used in this study are well-established, their application in shrimp gravlax products is still limited. The main contribution of this study lies in the integration of multiple quality indicators, including physicochemical, microbiological, and sensory parameters, within a unified machine learning framework. This approach enables a more comprehensive evaluation of product quality and supports practical shelf-life prediction by considering the combined effect of different spoilage mechanisms rather than relying on a single parameter.

In this context, the aim of the present study was to produce shrimp gravlax using a traditional curing process and to evaluate its shelf life through physicochemical, microbiological, and sensory analyses. In addition, machine learning techniques were applied to classify product freshness and to develop a predictive framework for shelf-life assessment.

## 2. Materials and Methods

### 2.1. Raw Material

The shrimp (*Penaeus japonicus*) used in the study were purchased fresh from the fish market in Elazığ province and brought to the laboratory of Firat University, Faculty of Fisheries Processing Technology, in the cold chain without direct contact with ice. Black pepper, dill, lemon, salt, and sugar used in the gravlax process were purchased from local markets. All chemicals used in the analysis of the study were obtained from Sigma and Aldrich (St. Louis, MO, USA). The chemicals used were of analytical purity.

### 2.2. Preparation of Shrimp Gravlax

**Figure 1:**
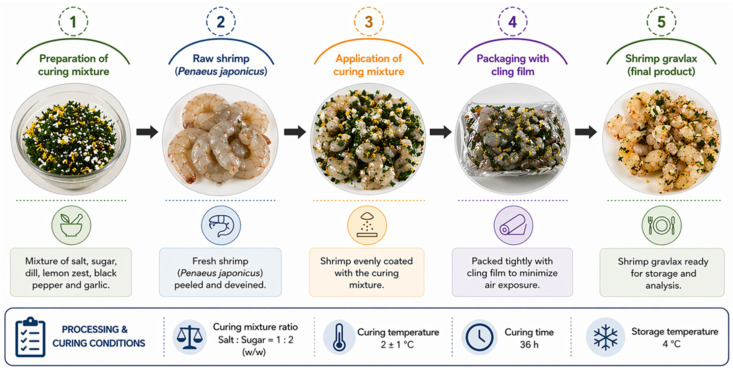
Schematicrepresentation of shrimp gravlax production process, including preparation of curing mixture, application to shrimp, packaging, and curing under controlled conditions (2 ± 1 °C for 36 h).

Shrimp gravlax was prepared following the methods of Emir Çoban et al. [10], with minor modifications. A total of 240 shrimps, delivered in three batches, were transported to the laboratory. Upon arrival, the shrimps were washed, deshelled, and frozen at −20 °C for 7 days. After thawing at 2 °C, they were subjected to the gravlax process. For curing, shrimps were coated with a mixture of sugar and salt (1:2, *w*/*w*), together with black pepper, dill, and lemon zest. A total of 200 g of curing mixture was applied per 1 kg of shrimp. The coated samples were wrapped with cling film and matured at 2 ± 1 °C for 36 h (Figure 1). During maturation, the accumulated liquid was drained at regular intervals, approximately every 12 h. At the end of the curing period, the residual curing mixture was gently removed from the shrimp surface using a kitchen brush. All curing conditions were kept constant throughout the study.

**Figure 2:**
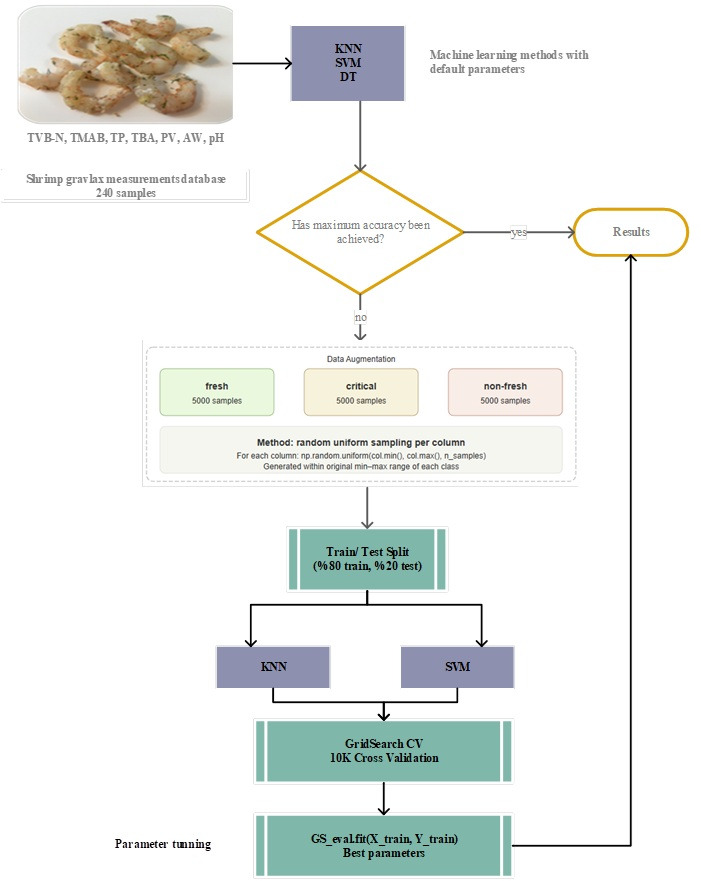
Proposed machine learning-based shelf-life control algorithm.

The gravlax shrimps were portioned into 100 g samples, vacuum-packed using a Henkelman Boxer 42 packaging machine (Henkelman Ind. Co., Boxmeer, The Netherlands), and stored at 4 °C for 30 days. Randomly selected samples were withdrawn every 5 days, as determined by preliminary trials, for physicochemical, microbiological, and sensory quality assessments. The resulting dataset was subsequently used for machine learning (ML) modeling. The experimental setup is shown in detail in Figure 2.

### 2.3. Physicochemical Analyses

pH was measured using a calibrated digital pH meter [11]. Water activity (aw) values were determined at room temperature using a water activity analyzer (Testo 650, Lenzkirch, Germany) [11]. Peroxide value (PV), which indicates primary lipid oxidation products, was determined following the method of Mattissek et al. [12]. TVB-N and TBARs analyses were carried out according to the method described by Varlık et al. [13]. TVB-N was determined using the distillation method, while TBARs values were expressed as mg malondialdehyde (MDA)/kg sample. All analyses were carried out in triplicate.

### 2.4. Microbiological Analyses

The numbers of total mesophilic aerobic bacteria (TMAB) and total psychrophilic bacteria (TP) were determined according to the methods reported by Halkman [14]. Ten grams of the shrimp gravlax sample were homogenized with 90 mL of 0.1% peptone water using a Stomacher (Model 400, Seward, London, UK) for 2 min. Serial decimal dilutions were prepared as required. Microbial counts were expressed as log colony-forming units per gram of sample (log CFU/g).

### 2.5. Sensory Analysis

Panelists evaluated shrimp gravlax samples in comparison with a control product (commercial salmon gravlax). A total of 42 panelists (aged 25–60), experienced in gravlax products and recruited from the Faculty of Fisheries at Fırat University, participated in the study. The number of panelists was determined based on the recommendations of Hough et al. [15].

Sensory evaluation was carried out under controlled laboratory conditions using individual booths to minimize bias. Samples were coded with random three-digit numbers and presented to panelists in a randomized order. Panelists evaluated the samples in terms of color, odor, flavor, and overall acceptability using a five-point hedonic scale (1 = dislike very much, 5 = like very much). Water and plain crackers were provided for palate cleansing between samples.

Participation in the sensory evaluation was voluntary, and panelists were informed about the study prior to evaluation. The study was conducted in accordance with general ethical principles for research involving human participants.

### 2.6. Machine Learning Modeling

Machine Learning (ML), which is a subfield of Artificial Intelligence (AI), allows systems to learn from data and make predictions without being explicitly programmed. ML methods are widely used in fields such as food science, medicine, finance, and engineering to improve efficiency and support decision-making processes [16,17]. In this study, three commonly used machine learning algorithms, namely Support Vector Machines (SVM), K-Nearest Neighbors (KNN), and Decision Trees (DT), were applied to determine the shelf life and freshness status of gravlax shrimp products.

At the initial stage of the study, a dataset consisting of 240 gravlax shrimp samples was created. For each sample, chemical parameters (TVB-N, TBA, PV), physicochemical parameters (pH and water activity, aw), and microbiological parameters (total mesophilic aerobic bacteria (TMAB) and total psychrophilic bacteria (TP)) were measured. The first measurements were carried out on raw shrimp, and subsequent analyses were performed at 5-day intervals during the storage period. The samples were classified as “fresh”, “critical”, or “not fresh” based on quality criteria reported in the literature [13]. In this classification, multiple physicochemical and microbiological parameters were evaluated together. Among these parameters, TVB-N was used as a reference indicator for defining freshness classes, since it is widely accepted as a reliable indicator of seafood spoilage. Accordingly, samples with TVB-N values below 25 mg/100 g were classified as “fresh”, those between 25 and 35 mg/100 g were classified as “critical”, and those above 35 mg/100 g were classified as “not fresh”. However, this classification was not based solely on TVB-N. All measured parameters, including TBA, PV, TMAB, TP, pH, and aw, were used together as input variables in the machine learning models, and the prediction of freshness classes was carried out by considering the combined effect of all quality indicators rather than relying on a single parameter.

The overall workflow of the proposed machine learning-based shelf-life prediction approach is presented in Figure 2. In the first step, the raw dataset was analyzed using KNN, SVM, and DT algorithms with their default parameter settings in order to obtain an initial reference for classification performance and to evaluate whether the available data were sufficient for accurate prediction.

Since the size of the experimental dataset was relatively limited (n = 240), a data augmentation step was applied to improve the robustness and generalization ability of the models. In this step, additional data points were generated for each freshness class using a random interpolation approach within the minimum and maximum values observed in the original dataset. During this process, the overall distribution of the original data was preserved and values outside the observed range were not generated. As a result, the number of samples in each class was increased to 5000, resulting in a total dataset of 15,000 samples, which enabled the models to learn from a broader range of variability while maintaining consistency with the original data.

In the final stage, the augmented dataset was divided into training and test sets with a ratio of 80% and 20%, respectively. Hyperparameter optimization was then performed for the KNN and SVM models using the GridSearchCV method, and model performance was systematically evaluated during this process. In addition, 10-fold cross-validation was applied to ensure the reliability of the results and to minimize the risk of overfitting. Based on these analyses, the model with the best-performing parameter combination was selected, and the freshness classification of gravlax shrimp samples was completed by achieving the highest possible classification accuracy under the given conditions.

### 2.7. Statistical Analysis

In the evaluation of the data obtained in the research, IBM SPSS^®^26 software (SPSS Inc., Chicago, IL, USA) was used. One-way analysis of variance (ANOVA) was applied to assess differences between groups (*p* < 0.05). A paired sample t-test was used to analyze the sensory data and compare panelists’ opinions between groups.

## 3. Results and Discussion

### 3.1. Physicochemical Changes During Storage

Changes in physicochemical properties of shrimp gravlax during storage at 4 °C are presented in Table 1. The results clearly show the effects of the gravlax process and subsequent storage on product quality.

The initial water activity (aw) of raw shrimp was 0.94 and decreased significantly to 0.79 after the gravlax process (*p* < 0.05), indicating a reduction in available water due to curing. During storage, aw values showed a slight increase and reached 0.83 at the end of the storage period. Despite this increase, aw values remained significantly lower than those of raw shrimp throughout storage (*p* <0.05), suggesting that the product maintained reduced water availability. Similar reductions in aw due to salting have been reported by Pongsetkul et al. [11].

The TVB-N value of raw shrimp was 18.53 mg/100 g and significantly decreased after processing (*p* < 0.05), which may be attributed to the diffusion of nitrogenous compounds during curing. During storage, TVB-N values increased steadily and reached 33.92 mg/100 g on day 30. This increase indicates progressive spoilage, and the values approached the commonly accepted limit after the 25th day. Comparable increases in TVB-N during storage of gravlax products have been reported by Altan et al. [7] and Yıldız and Kızıloğlu [18].

TBARs values decreased from 0.34 to 0.11 mg MDA/kg after the gravlax process, indicating improved oxidative stability. During storage, TBAR values increased gradually and reached 4.12 mg MDA/kg on day 30. Although this value remained below the critical threshold reported in the literature [13], a marked increase after day 25 suggests ongoing lipid oxidation. Similar trends have been observed in gravlax products stored under refrigerated conditions [2,19].

Peroxide values (PVs) followed a similar pattern, decreasing after processing and then increasing during storage. At the end of the storage period, PV reached 6.23 meq/kg. Despite this increase, PV remained within acceptable limits, indicating that primary lipid oxidation was controlled throughout storage. These findings are consistent with previous studies reporting gradual increases in PV during storage of cured seafood products [18,19].

The pH value of raw shrimp was 7.56 and decreased to 7.01 after processing. During storage, pH values increased gradually and reached 8.13 at the end of the storage period. This increase is likely associated with the formation of basic compounds such as ammonia during spoilage. Similar pH changes have been reported in gravlax and other cured fish products [2,5].

Overall, the combined evaluation of physicochemical parameters indicates that shrimp gravlax maintained acceptable quality up to approximately 25 days under refrigerated storage conditions.

**Figure 3:**
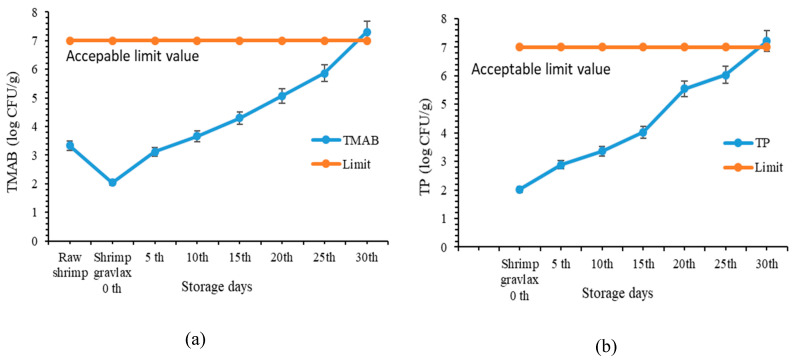
Changes in (**a**) total mesophilic aerobic bacteria (TMAB) and (**b**) total psychrophilic bacteria (TP) counts of shrimp gravlax during storage at 4 °C.

Changes in microbial counts of shrimp gravlax during storage at 4 °C are presented in Figure 3. The initial total mesophilic aerobic bacteria (TMAB) and total psychrophilic bacteria (TP) counts of raw shrimp were 3.33 and 2.58 log CFU/g, respectively. After the gravlax process, both TMAB and TP levels decreased significantly (*p* < 0.05), indicating that the curing ingredients effectively inhibited microbial growth.

During storage, TMAB and TP counts increased gradually. The increase in microbial load became more pronounced after day 20. This trend indicates progressive microbial growth under refrigerated conditions and suggests that microbial spoilage becomes significant after this period. Similar increases in microbial counts during storage of gravlax products have been reported by Namiq and Milne [20] and Ozpolat [21], confirming that microbial growth continues even under refrigerated conditions.

Overall, the microbiological results support the physicochemical findings and indicate that shrimp gravlax maintained acceptable microbiological quality  (7 log CFU/g) up to approximately 25 days under refrigerated storage conditions.

### 3.2. Sensory Evaluation

**Figure 4:**
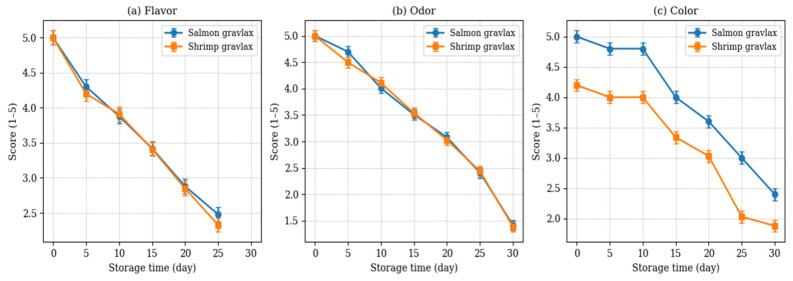
Changes in (**a**) flavor, (**b**) odor, and (**c**) color scores of salmon and shrimp gravlax during storage at 4 °C. Values are expressed as mean ± standard deviation.

Changes in the sensory properties of shrimp gravlax during storage at 4 °C are shown in Figure 4. At the beginning of storage, shrimp gravlax received high scores for flavor, odor, and color. As storage progressed, these scores gradually decreased, indicating a decline in overall quality. Among the evaluated attributes, odor showed the most noticeable change and reached the lowest scores by the end of the storage period, while flavor and color were relatively more stable. When compared with salmon gravlax, no significant differences were found in flavor and odor (*p* > 0.05). However, salmon gravlax was rated higher in terms of color (*p* < 0.05), most likely due to its more intense appearance. The lighter color of shrimp gravlax may have influenced panelist preferences in this regard. Despite the decrease during storage, sensory scores remained at acceptable levels up to approximately day 25. After this point, a clearer decline in overall acceptability was observed. This trend is in line with the physicochemical and microbiological results. Panelists also noted that the combination of dill, salt, sugar, lemon zest, and black pepper contributed positively to the flavor of shrimp gravlax. Similar effects of seasoning on sensory quality have been reported in previous gravlax studies [22,23,24].

### 3.3. Shelf-Life Assessment Using Machine Learning

The performance of the machine learning models used for the classification of shrimp gravlax samples was evaluated using accuracy, precision, recall, and F1-score metrics. These metrics provide a comprehensive assessment of classification performance from different perspectives. The mathematical expressions of these metrics are given in Equations (1)–(4).

(1) $$Accuracy=\frac{TP+TN}{TP+TN+FP+FN}$$

(2) $$\mathrm{Pr}ecision=\frac{TP}{TP+FP}$$

(3) Recall=TPTP+FN

(4) $$F1-Score=\frac{2TP}{2TP+FP+FN}$$

The classification results obtained using default parameters are presented in Table 2.

As shown in Table 2, the SVM model achieved an accuracy of 95.83%, the K-NN model achieved 97.22%, and the DT model reached 100% accuracy. These results indicate that the gravlax dataset can be classified with high accuracy, particularly using tree-based methods. Similar findings have been reported in the literature, where SVM and DT models demonstrate strong performance in food quality prediction tasks [4,5].

**Figure 5:**
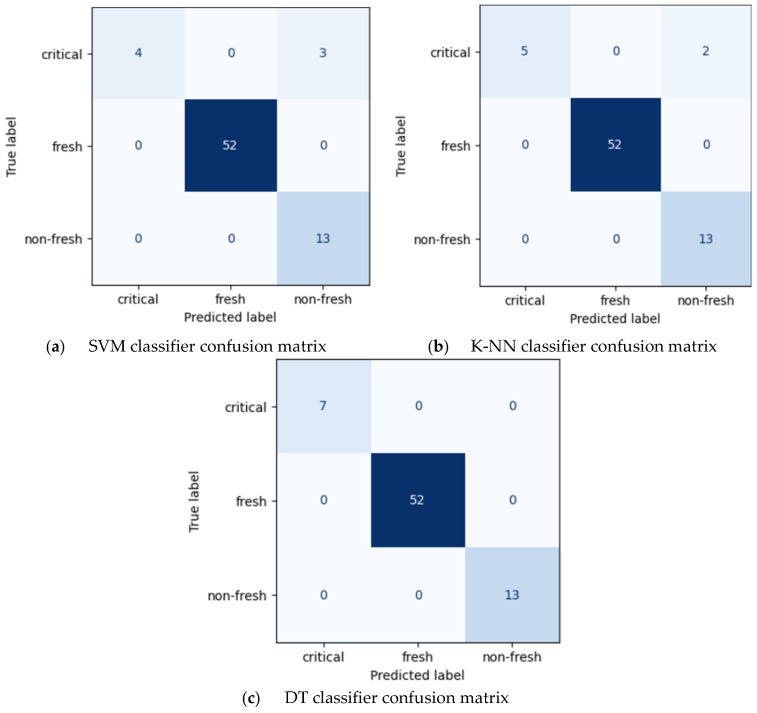
Confusion matrix of proposed classifiers with default parameters.

The confusion matrices corresponding to the classification results obtained with default parameters are presented in Figure 5.

As illustrated in Figure 5, most samples were correctly classified, while misclassifications were mainly observed between adjacent classes. This indicates that the models were able to effectively distinguish between different freshness levels. In order to improve model performance, a data augmentation approach was applied, followed by hyperparameter optimization using GridSearchCV. For the SVM model, the optimal parameters were determined as C = 100 with an “rbf” kernel function. For the K-NN model, the optimal number of neighbors was found to be K = 3 using the Manhattan distance metric. The classification results obtained after hyperparameter optimization are presented in Table 3.

**Figure 6:**
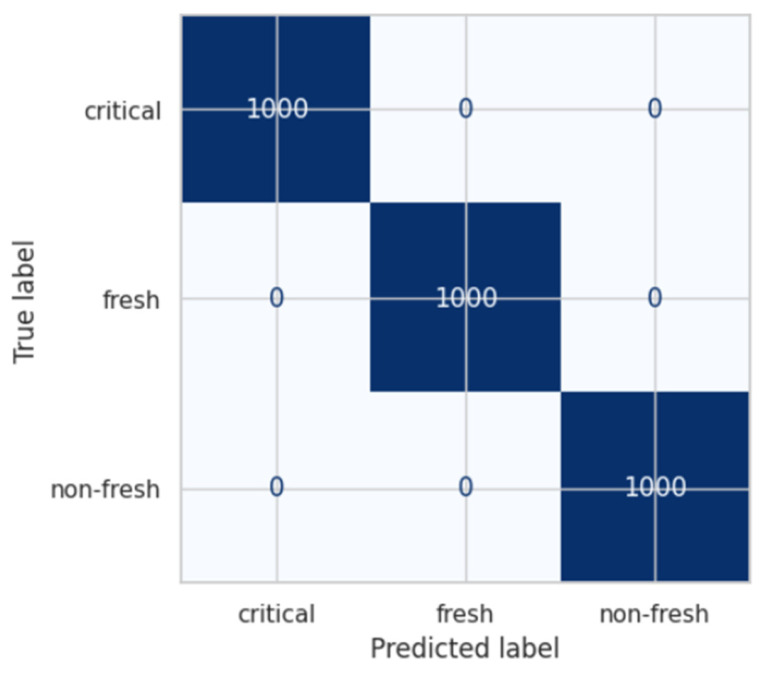
Finalconfusion matrix of SVM and K-NN classifiers.

As shown in Table 3, both SVM and K-NN models achieved 100% accuracy after optimization. This result indicates that data augmentation and parameter tuning significantly improved model performance. The final confusion matrix results obtained after optimization are presented in Figure 6.

As illustrated in Figure 6, all samples were correctly classified, and no misclassification was observed. This demonstrates that model performance can be substantially improved through dataset expansion and proper parameter optimization.

The model predictions were found to be consistent with the experimental findings. In particular, the transitions between freshness classes were in agreement with the changes observed in physicochemical, microbiological, and sensory parameters during storage. Previous studies have also reported that machine learning approaches can provide reliable results for shelf-life prediction in food systems [25,26,27].

Considering all results together, the shelf life of shrimp gravlax was estimated to be approximately 25 days under the storage conditions applied in this study.

However, it should be noted that all experiments were carried out under a single storage condition (4 °C), which is a limitation of the study. In addition, the dataset is based on measurements taken from the same batches over time, which may introduce some level of data correlation. The models developed in this study were intended to classify freshness at specific time points rather than to predict future changes. Therefore, the results mainly reflect the conditions used in this study. Future studies including different storage conditions, independent datasets, and time-based modeling approaches, especially those using early-stage data to estimate later changes, would help to improve the applicability of the model.

## 4. Conclusions

In this study, the application of the gravlax process to shrimp resulted in a product with reduced water activity and improved initial stability, indicating that curing methods can be successfully adapted to shrimp as a ready-to-eat alternative. The combined effects of salt, sugar, and controlled storage conditions contributed to slowing down microbial growth and biochemical deterioration during refrigerated storage.

The results clearly showed that the shelf life of shrimp gravlax was limited by the gradual increase in protein degradation, lipid oxidation, and microbial activity, which became more pronounced after approximately 25 days. The consistency observed between physicochemical, microbiological, and sensory findings highlights the importance of using multiple quality indicators for reliable shelf-life assessment.

From a methodological perspective, the study demonstrated that machine learning models can effectively classify product freshness when multiple parameters are considered together. Rather than relying on a single indicator, the integration of different quality attributes provided a more robust evaluation framework. This approach may be particularly useful in complex food systems where quality changes are influenced by multiple interacting factors.

However, it should be noted that the models developed in this study were based on data obtained under a single storage condition and were not designed to predict future changes over time. Therefore, their applicability is limited to classification within the studied conditions. Future studies focusing on independent datasets and time-dependent modeling approaches would further enhance the practical use of machine learning in shelf-life prediction.

Overall, the findings suggest that shrimp gravlax represents a viable ready-to-eat seafood product and that combining traditional processing techniques with data-driven methods can provide valuable support for quality monitoring and shelf-life evaluation.

## Figures and Tables

**Table 1:** Physicochemical properties of raw shrimp and gravlax prepared from shrimp.

Samples		pH	aw	TVB-N (mg/100 g)	TBARs (mg MDA/kg)	PVs (meq/kg)
Raw shrimp		7.56 ± 0.02 ^c^	0.94 ± 0.07 ^a^	18.53 ± 0.06 ^e^	0.34 ± 0.09 ^g^	0.98 ± 0.12 ^g^
Shrimp gravlax	0th	7.01 ± 0.11 ^d^	0.79 ± 0.07 ^e^	12.44 ± 0.04 ^g^	0.11 ± 0.05 ^h^	0.60 ± 0.09 ^h^
	5th	7.08 ± 0.05 ^d^	0.79 ± 0.05 ^e^	15.87 ± 0.03 ^f^	0.76 ± 0.14 ^f^	1.49 ± 0.14 ^f^
	10th	7.65 ± 0.06 ^cd^	0.82 ± 0.02 ^cd^	18.88 ± 0.02 ^e^	1.14 ± 0.11 ^e^	3.55 ± 0.04 ^e^
	15th	7.80 ± 0.03 ^b^	0.80 ± 0.01 ^d^	20.46 ± 0.05 ^d^	2.02 ± 0.13 ^d^	4.82 ± 0.10 ^d^
	20th	8.08 ± 0.08 ^a^	0.82 ± 0.06 ^cd^	24.05 ± 0.04 ^c^	2.55 ± 0.22 ^c^	5.08 ± 0.04 ^c^
	25th	7.94 ± 0.15 ^ab^	0.82 ± 0.03 ^c^	29.12 ± 0.09 ^b^	3.74 ± 0.10 ^b^	5.67 ± 0.24 ^b^
	30th	8.13 ± 0.09 ^a^	0.83 ± 0.03 ^b^	33.92 ± 0.03 ^a^	4.12 ± 0.08 ^a^	6.23 ± 0.20 ^a^

The results are represented as the means ± standard deviation (n = 3); Values within each column with different lowercase letters are significantly different (*p* < 0.05).

**Table 2:** Classification results with default parameters.

Metric (%)	SVM	K-NN	DT
Accuracy	95.83	97.22	100
Precision	94.0	96.0	100
Recall	86.0	90.0	100
F1-Score	88.0	92.0	100

**Table 3:** Classification results after hyperparameter optimization.

Metrics (%)	SVM	K-NN
Accuracy	100	100
Precision	100	100
Recall	100	100
F1-Score	100	100

## Data Availability

The original contributions presented in this study are included in the article. Further inquiries can be directed to the corresponding author.
